# Insulin increases type I collagen synthesis in hepatic stellate cells via α5β1 integrin

**DOI:** 10.20517/mtod.2024.59

**Published:** 2024-12-10

**Authors:** Milan Dodig, Min Li, Srinivasan Dasarathy, Sivarajan Kumarasamy, Takhar Kasumov, Sonia M. Najjar, Arthur J McCullough

**Affiliations:** 1 Division of Gastroenterology and Hepatology, Cleveland Clinic, Cleveland, OH 44195, USA.; 2 Department of Biomedical Sciences and Diabetes Institute, Heritage College of Medicine, Ohio University, Athens, OH 45701, USA.; 3 Department of Pharmaceutical Sciences, Northeast Ohio Medical University, Rootstown, OH 44272, USA.

**Keywords:** Collagen, insulin, hepatic stellate cells, insulin resistance, α5β1 integrin

## Abstract

**Aim:**

A direct effect of insulin on the synthesis of extracellular matrix proteins has been described in extrahepatic organs. The current study investigates the role of insulin in type I collagen production in hepatic stellate cells (HSCs).

**Methods:**

Primary HSC cultures from wild-type mice and from L-SACC1 transgenic mice that exhibit hyperinsulinemia and resultant insulin resistance due to a defect in hepatic insulin clearance were used.

**Results:**

Insulin significantly increased type I collagen synthesis in HSC primary cultures in the presence of high but not low glucose concentrations. Although HSCs contain a functional, insulin-activated PI3 kinase signaling pathway, insulin increases type I collagen synthesis by mechanisms independent of PI3 kinase. Insulin stimulated α5β1 integrin levels and phosphorylation of focal adhesion kinase, a major signaling mediator in the integrin pathway. In addition, α5β1 integrin siRNA interference abolished insulin-mediated type I collagen synthesis by HSCs. L-SACC1 mice showed increased hepatic collagen deposition as compared to wild-type mice. HSCs isolated from L-SACC1 mice synthesize more type I collagen and α5β1 integrin than HSCs isolated from wild-type controls.

**Conclusion:**

Insulin exerts a direct profibrotic impact on HSCs by an α5β1 integrin-mediated mechanism, independently of the PI3 kinase signaling pathway. Thus, chronic hyperinsulinemia may potentiate liver collagen deposition in insulin resistance states. This likely increases the risk of significant fibrosis burden in chronic liver disease associated with insulin resistance.

## INTRODUCTION

Patients with type 2 diabetes mellitus (T2DM) have increased liver-related morbidity and mortality. T2DM is characterized by insulin resistance and chronically elevated high levels of circulating insulin. T2DM is an independent predictor of fibrosis in metabolic dysfunction-associated steatohepatitis (MASH)^[1]^, as well as an increasing risk of chronic liver disease and hepatocellular carcinoma^[2]^. Additionally, insulin resistance, hyperinsulinemia, and T2DM have been associated with the progression of hepatic fibrosis in hepatitis C patients^[3,4]^. Accumulating evidence suggests that the complications of diabetes may precede its clinical manifestations, emphasizing the critical role of insulin resistance and hyperinsulinemia in the pathogenesis of T2DM-related morbidity^[5]^.

Hyperinsulinemia increases type I collagen content in skeletal muscle in obese subjects and in patients with diabetes^[6]^. Even in healthy individuals, experimentally induced insulin resistance stimulates extracellular matrix (ECM) gene expression in striated muscle, including type I collagen and fibronectin^[7]^. Moreover, several animal models of insulin resistance exhibit altered ECM and increased fibrosis in the aorta, heart, and kidney^[8–10]^. Evidence of increased hepatic fibrosis in animal models of MASH in the background of insulin resistance and diabetes is mounting^[11–14]^. Whether hyperinsulinemia associated with insulin resistance plays a direct role in fibrosis remains unclear^[15]^.

Although insulin modifies ECM in various extrahepatic tissues and cell models^[16,17]^, reports of a direct insulin effect on the activation of hepatic stellate cells (HSCs) and matrix secretion are inconclusive. Thus, the current study was undertaken to address whether insulin can directly activate HSCs. Because HSCs are the main source of hepatic type I collagen production following liver injury, we used an established model of primary HSC cultures^[18]^ to test the hypothesis that high levels of insulin exert a direct profibrotic effect on HSCs by inducing collagen synthesis. To further examine the effect of chronically elevated levels of insulin on the development of liver fibrosis *in vivo*, we used the L-SACC1 transgenic mouse model of hyperinsulinemia without fasting hyperglycemia. This mouse harbors a liver-specific dominant negative Ser503Ala mutation of the carcinoembryonic antigen-related cell adhesion molecule 1 (CEACAM1). CEACAM1 is a plasma membrane glycoprotein that is expressed as two alternatively spliced variants. The long isoform (CEACAM1–4L) contains a longer 71 amino acid-intracellular tail, which includes tyrosine and serine phosphorylation sites that are lacking in the short isoform^[19]^. Among these sites is Ser503, which contributes to targeting the long isoform to the sinusoidal domain of hepatocytes^[20]^, where it promotes receptor-mediated insulin uptake for subsequent degradation and clearance^[21]^. The Ser503Ala mutation disrupts this cellular mechanism, impairing hepatic insulin clearance and thus leading to chronic hyperinsulinemia and ensuing insulin resistance, but without fasting hyperglycemia^[15]^. In L-SACC1 mice, hyperinsulinemia results from impaired hepatic insulin clearance rather than a primary defect in insulin signaling or response to increased visceral obesity^[15]^. As expected from spontaneous hepatic fibrosis that develops in these mice^[11]^, we herein show that the L-SACC1 mouse exhibits an increase in the amount of collagen-based ECM in the liver under basal conditions. Moreover, HSCs derived from this mouse show higher synthesis of type I collagen. These observations provide mechanistic clues on the profibrotic effect of hyperinsulinemia in the liver and emphasize its critical role in the pathogenesis of metabolic dysfunction-associated steatotic liver disease (MASLD) in humans^[22]^ and mice^[12]^.

## METHODS

### Studies in HSCs

HSCs were isolated from transgenic mice harboring 3.6 kb of rat COL1A1 type I collagen promoter linked to the chloramphenicol acetyl-transferase (CAT) reporter gene^[16]^, as well as L-SACC1 and wild-type controls^[23]^, as routinely done^[24]^. Transgenic mice and measurements of the CAT reporter gene were used to focus on changes in COL1A1 gene expression independent of any post-transcriptional and post-translational processing involved in type I collagen deposition by HSCs. Shortly after the animals were killed, livers were removed, minced, and digested in pronase-collagenase mixture. HSCs were separated from the resulting digest by gradient centrifugation in Nycodenz and plated in Dulbecco’s modified Eagle Medium (DMEM) with 10% fetal calf serum and 10% horse serum (Hyclone, Logan, UT) for the duration of the experiment with daily media changes. The purity of primary HSC cultures was confirmed by immunostaining for markers of HSC phenotype: α-smooth muscle actin (α-SMA) (Sigma, St. Louis, MO), nestin, and glial fibrillary acidic protein (GFAP), using commercially available antibodies.

### Insulin treatment

Primary cultures were treated with insulin (Sigma, St. Louis, MO) from day 2–7 of culture. Initial experiments showed that insulin increased type I collagen production by HSCs in a wide range of concentrations (from 0.05–50 μmol). We chose to use 5 μmol in all our subsequent experiments, as this dose consistently produced the strongest effect.

### CAT assay

Cells were scraped from primary HSC cultures in 0.3 mL of extraction buffer (0.25 M Tris-HCl, pH 7.8, containing 0.5% Triton X-100), subjected to three cycles of freezing and thawing and then heated at 65 °C for 15 min to inactivate endogenous deacetylases. After centrifugation, the supernatants were used to determine CAT activity and protein concentration. CAT activity was measured using a modified fluor diffusion assay^[16]^ and normalized to the protein content of the extract as measured by the BCA assay (Pierce, Rockford, IL).

### Proliferation assay

The proliferation of HSCs was measured with Alamar blue^®^ (Serotec, Westbury, NY), according to the manufacturer’s recommendations. Nine hundreds μL of fresh medium and one hundred μL of Alamar blue^®^ were added to 12-well cell culture plates, mixed gently and incubated for 4 h. Cell proliferation was determined by a colorimetric change in the medium measured as a difference in culture media absorbance at 570 and 600 nm. Like other studies^[17]^, we previously validated this method and showed a strong correlation with the cell number and BrdU incorporation.

### Protein isolation and western blot analysis

Day 7 primary HSC cultures were lysed with ice-cold lysis buffer containing 25 mM Tris pH 7.4, 50 mM NaCl, 25 mM NaF, 10% glycerol, 1% Triton-X100, PMSF (1 mM), protease inhibitor cocktail 10 μL/mL (Sigma #P8340, Sigma, St. Louis, MO), scraped and left on ice for 30 min. To detect phosphorylated AKT and focal adhesion kinase (FAK), cells were incubated in serum-free media for 24 h before treatment (day 6–7). Cultures were treated with insulin for 15, 30, and 60 min for pAKT analysis, and for 60 min for pFAK. Protein concentration was determined by detergent compatible (DC) protein assay (Biorad, Hercules, CA). Equal amounts of protein samples were resolved on a 7.5% SDS-PAGE under reducing conditions and then electro-transferred onto the polyvinylidene fluoride (PVDF) membrane. Antibodies used were as follows: α5 integrin (Santa Cruz Biotechnology), type I collagen (sc-8788, Santa Cruz, CA, USA), α-actin (Santa Cruz Biotechnology), AKT, p-AKT, FAK, and p-FAK (all purchased from Cell Signaling Technologies, Beverly, MA), and α5β1 integrin (Chemicon, Temecula, CA). After transfer, the membrane was blocked with 5% nonfat milk, 2% BSA in TBS with 0.1% Tween, incubated with goat α5β1 polyclonal antibody (Chemicon, Temecula, CA), washed, and then incubated with appropriate horseradish peroxidase (HRP)-conjugated secondary antibody (all purchased from Santa Cruz Biotechnology Inc, Santa Cruz, CA). The blots were visualized with the ECL detection system (Amersham, Piscataway, NJ).

### α5 integrin gene silencing in HSC cultures

HSCs were isolated as described above. Twenty-four hours after plating, HSCs were transfected with 10 nM of α5 integrin siRNA in HiPerFect transfection reagent. Cells were harvested and analyzed on day 5 of culture. Type I collagen and α5 integrin protein levels were determined in cell lysates after treatment with insulin or control buffer. Both α5 integrin siRNA and transfection reagent were purchased from Qiagen Inc. (Valencia, CA) and used according to the manufacturer’s recommendations.

### Cell adhesion assay

Cell Culture Cluster plates (96 wells) were coated with purified fibronectin (10 μg/mL in PBS) at 4 °C overnight. HSC cells were seeded at a concentration of 2.5 × 10^5^ per well with 100 μL of DMEM with 10% fetal calf serum and 10% horse serum. After 30-min incubation in the 5% CO_2_ incubator at 37 °C, unattached cells and culture medium were simultaneously removed by pipette, followed by washing with DMEM serum-free medium. Cells were then fixed in 1% glutaraldehyde in PBS, stained with 0.1% crystal violet, and lysed with 0.5% Triton X-100. Absorbance was measured at 595 nm in a plate reader.

### RNA isolation and semi-quantitative real time-PCR

Total RNA was isolated using TRI reagent (Sigma). Reverse transcriptase kits (Invitrogen, Carlsbad, CA) were used to generate cDNA per the manufacturer’s recommendations. Specific primers were designed and analyzed using commercial software (Light Cycler Probe Design, Roche Diagnostics). Primer sequences for α5 integrin are as follows: α5β1-forward primer: 5’-ACCAAGACGGCTACAATGATG-3’ and α5β1-reverse primer 5’-CTGCTTGGAAGTCAGGAACAG-3’. The GAPDH primers are as follows: GAPDH forward 5’-GATGACATCAAGAAGGTGGTGA-3’ and GAPDH reverse primer 5’-GGTCCAGGGTTTCTTACTCCTT-3’. Primers for the COL1A1 gene were COL1A1 forward 5’-ATGTTCAGCTTTGTGGACCTC-3’ and COL1A1 reverse 5’-AGTTTGAAGCACAGCACTCG-3’. Real-time PCR for quantification of RNA was carried out using the SYBR protocol on the fluorescence temperature cycler (Light Cycler; Roche Molecular Diagnostics, Indianapolis, IN). The reaction conditions were optimized at different temperature ranges and magnesium concentrations. Real-time reactions were carried out in duplicate, and amplicons were analyzed by generating melting curves with continuous measurement of fluorescence. Results were calculated as relative differences in target threshold cycle values normalized to GAPDH. All real-time PCR products were separated on a 1.5% Tris-acetic acid agarose gel to confirm product presence and size.

### Immunostaining of liver sections

α-SMA and GFAP antibodies for immunofluorescence were purchased from Sigma-Aldrich (St. Louis, MO), nestin antibody from Abcam (Cambridge, MA ) and Insulin Receptor Substrate-1 (IRS1) antibody from Santa Cruz (Santa Cruz, CA). HSCs were grown on slides and fixed in 3.7% formaldehyde in PBS. Slides were incubated in primary antibody overnight at 4 °C. After incubating with a secondary antibody labeled with a fluorophore (Invitrogen, Carlsbad, CA), immunofluorescence was visualized by fluorescent microscope. For negative controls, we used serum corresponding to the primary antibody source.

### Sirius red staining of liver sections

The collagen matrix in livers from L-SACC1 and wild-type mice was visualized and quantified with Sirius red staining. The liver was diced into 5X5-mm sections, immersion-fixed in PBS containing 10% Formalin for 24 h, and embedded in paraffin. 10 μm sections were mounted on glass slides, deparaffinized and the slides rehydrated as follows with a wash for each 3-min-step: Xylene substitute (three times), 100% ethyl alcohol (EtOH) (twice), 70% EtOH, 30% EtOH, and 0.5% Tween20 in PBS (twice). Sections were incubated for 1 h at room temperature with an aqueous solution of saturated picric acid containing 0.1% direct red 80 (Sigma-Aldrich) and covered with aluminum foil during the incubation. Stained slides were washed with 0.5% glacial acetic acid solution 3 times (5 min each), dehydrated (3 min each step), mounted, and examined using polarized light under a light microscope. Image-Pro Plus^®^ software (Media Cybernetics, Silver Spring, MD) was performed to calculate the percentage of Sirius red staining. The percent volume of collagen (C_p_) was calculated according to the equation C_p_ = (A_c_/A_b_) X100 % (where A_c_ equals the area of collagen and A_b_ equals the total area). Comparisons between the wild-type and transgenic groups were conducted using *t*-test for unequal variance, where *P* < 0.05 is indicative of statistical significance.

## RESULTS

### The effect of insulin on collagen production in HSCs

**Figure 1.**
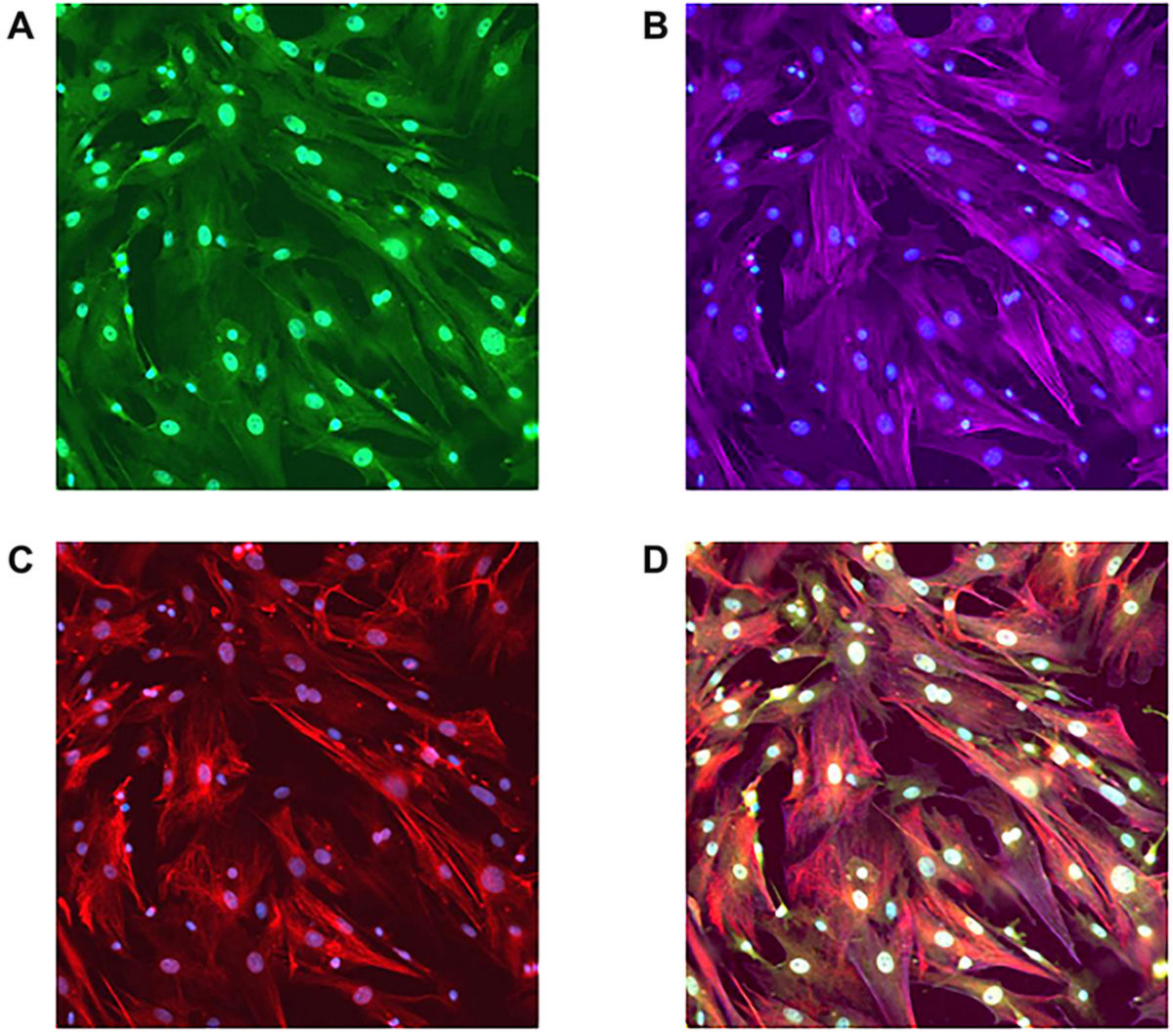
Purity of primary hepatic stellate cell cultures. Primary HSC cultures on day 7 were immunostained for markers of stellate cell phenotype: GFAP (A), α-SMA (B) and nestin (C). The composite image (D) of the triple-stained slide shows that HSCs were stained by all three markers, indicating their purity. HSCs: Hepatic stellate cells; GFAP: glial fibrillary acidic protein; α-SMA: alpha-smooth muscle actin.

Primary hepatic stellate cell cultures were grown for 7 days, and their purity was assessed by immunostaining for stellate cell markers [Figure 1]. Representing triple immunofluorescence for GFAP [Figure 1A], α-SMA [Figure 1B], and nestin [Figure 1C], the majority of cells in culture stained for all three markers (seen best at composite image Figure 1D).

**Figure 2.**
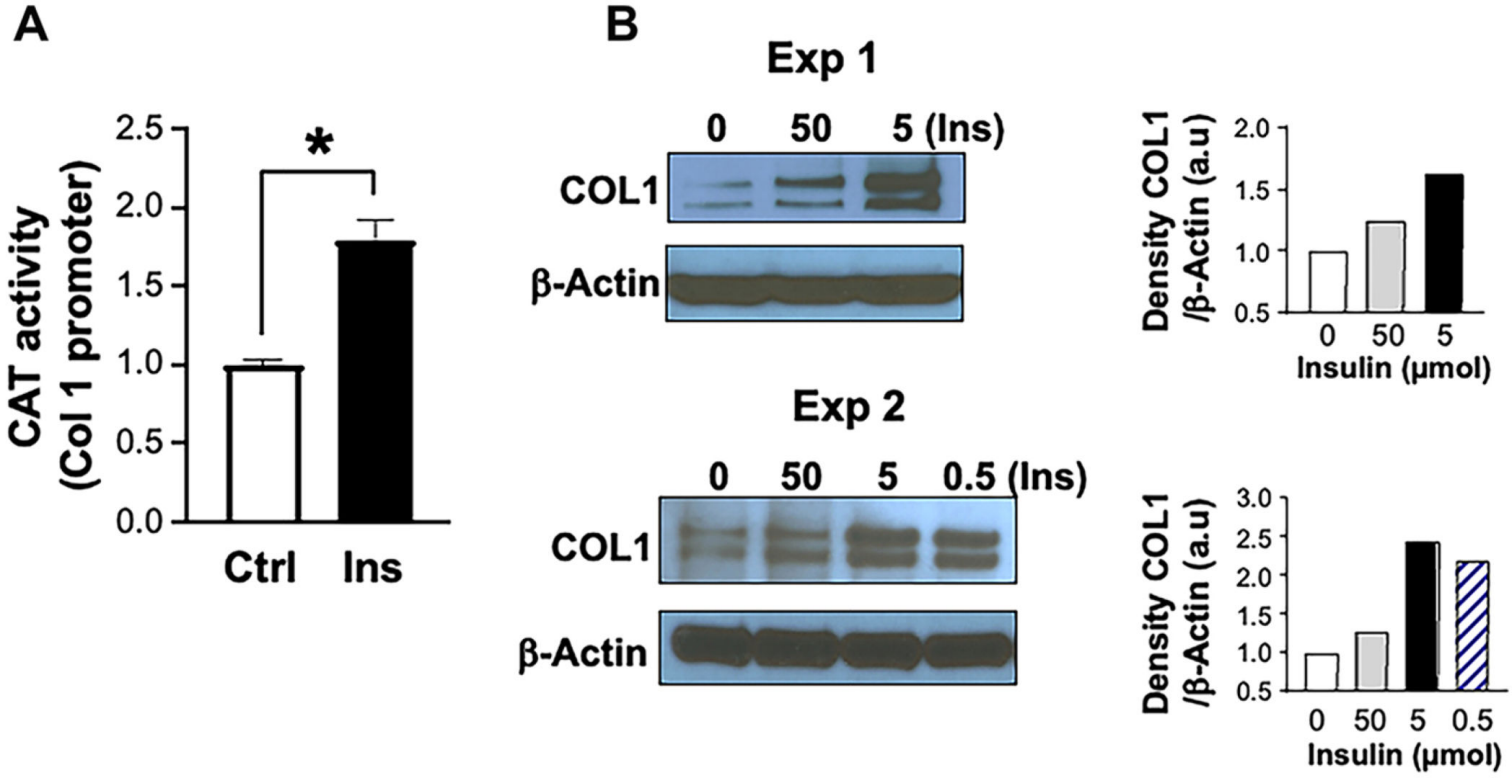
The insulin effect on type 1 collagen production. (A) represents higher type I collagen (COL1) in the presence of insulin (Ins, 5 μmol) relative to its absence (Ctrl). Results are compiled from 4 independent experiments. Average Ctrl CAT activity is shown as 1 (100%), with bars representing SEM; (B) represents Western blot analysis of COL1 protein levels following treatment with Ins from two independent experiments. HSC cultures were treated with different doses of Ins ranging from 0.5–50 μmol. All tested doses increased the amount of synthesized COL1, with 5 μmol being the most effective. β-actin was used for normalization against loaded proteins. Gels were scanned using image J v1.53t, and the density of each test band was divided by its corresponding loading Ctrl, represented in arbitrary units (a.u) in the accompanying graph. CAT: Chloramphenicol acetyltransferase; HSCs: hepatic stellate cells; COL1: type I collagen; Ins: insulin; Ctrl: control; SEM: standard error of mean.

Treating primary HSC cultures with insulin (5 μmol) from days 2–7 significantly increased type I collagen promoter activity, as assessed by CAT assay [Figure 2A]. Moreover, Western blot analysis showed increased type I collagen protein levels in response to a wide range of insulin concentrations [Figure 2B]. Thereafter, insulin at 5 μmol concentration was used since it exerted a profound effect, although even 50 nmol exerted a significant effect (data not shown).

**Figure 3.**
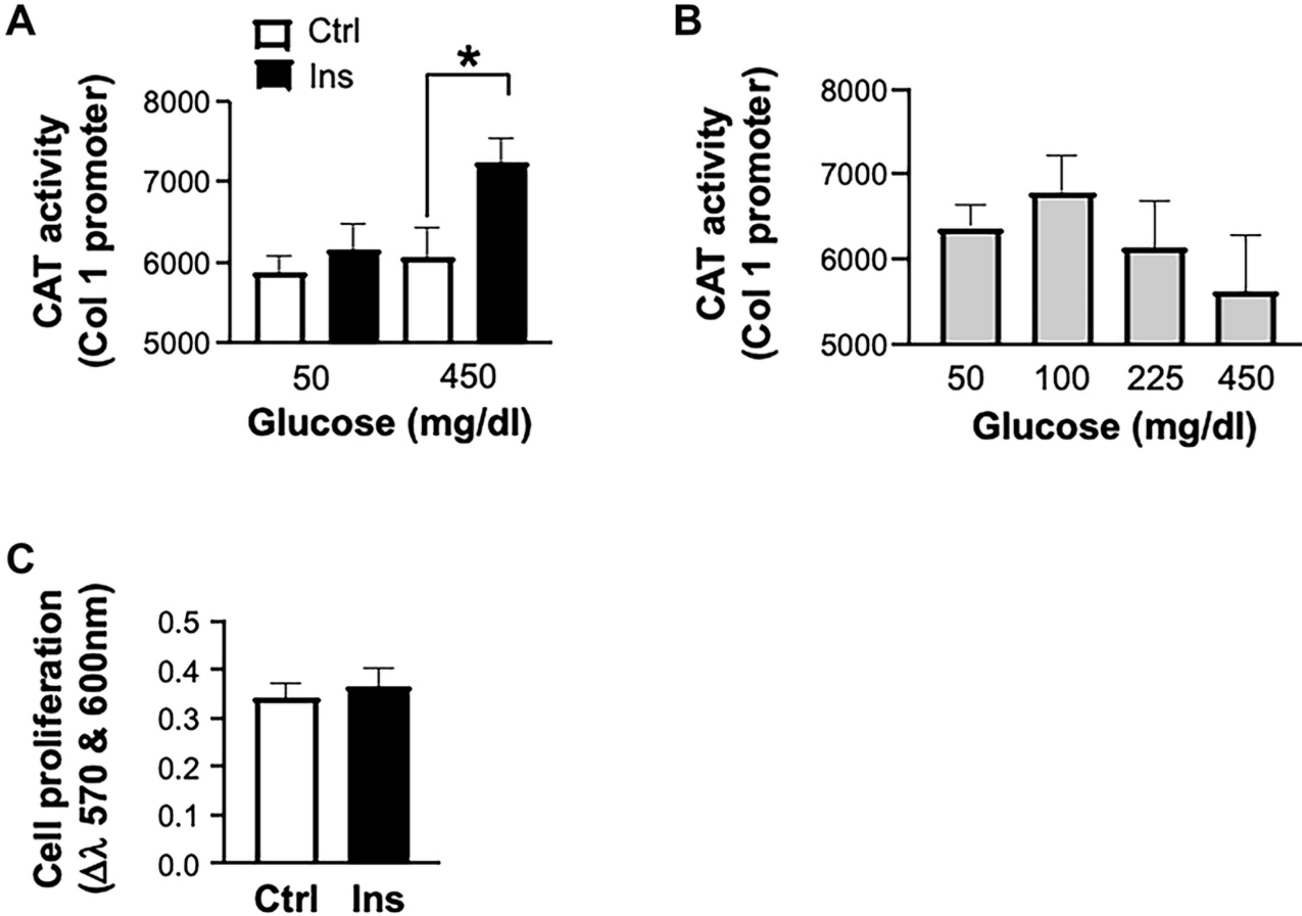
The effect of glucose and insulin on type I collagen synthesis and HSCs proliferation. Primary HSC cultures were incubated in DMEM enriched with 50 or 450 mg/dL glucose with (Ins, 5 μmol) or without (Ctrl) Ins. COL1 promoter activity was assayed as in the legend in Figure 2A (A). Promoter activity of COL1 was assayed in cells incubated with different glucose concentrations (in mg/dL, X-axis) without concomitant Ins treatment (B). Alamar blue assay was used to test the effect of Ins on cell proliferation. Cell proliferation was determined by a colorimetric change in the medium measured as a difference in culture media absorbance at 570 and 600 nm (C). HSCs: Hepatic stellate cells; DMEM: Dulbecco’s modified Eagle Medium; COL1: type I collagen; Ins: insulin; Ctrl: control.

The insulin effect was observed when HSCs were cultured in DMEM containing high glucose (450 mg/dL) but not low glucose concentrations (50 mg/dL) [Figure 3A]. However, glucose over a wide range of 50–450 mg/dL did not upregulate type I collagen promoter activity in the absence of insulin [Figure 3B]. Interestingly, the proliferation of HSCs was not affected by insulin treatment [Figure 3C].

### Insulin signaling in HSCs

**Figure 4.**
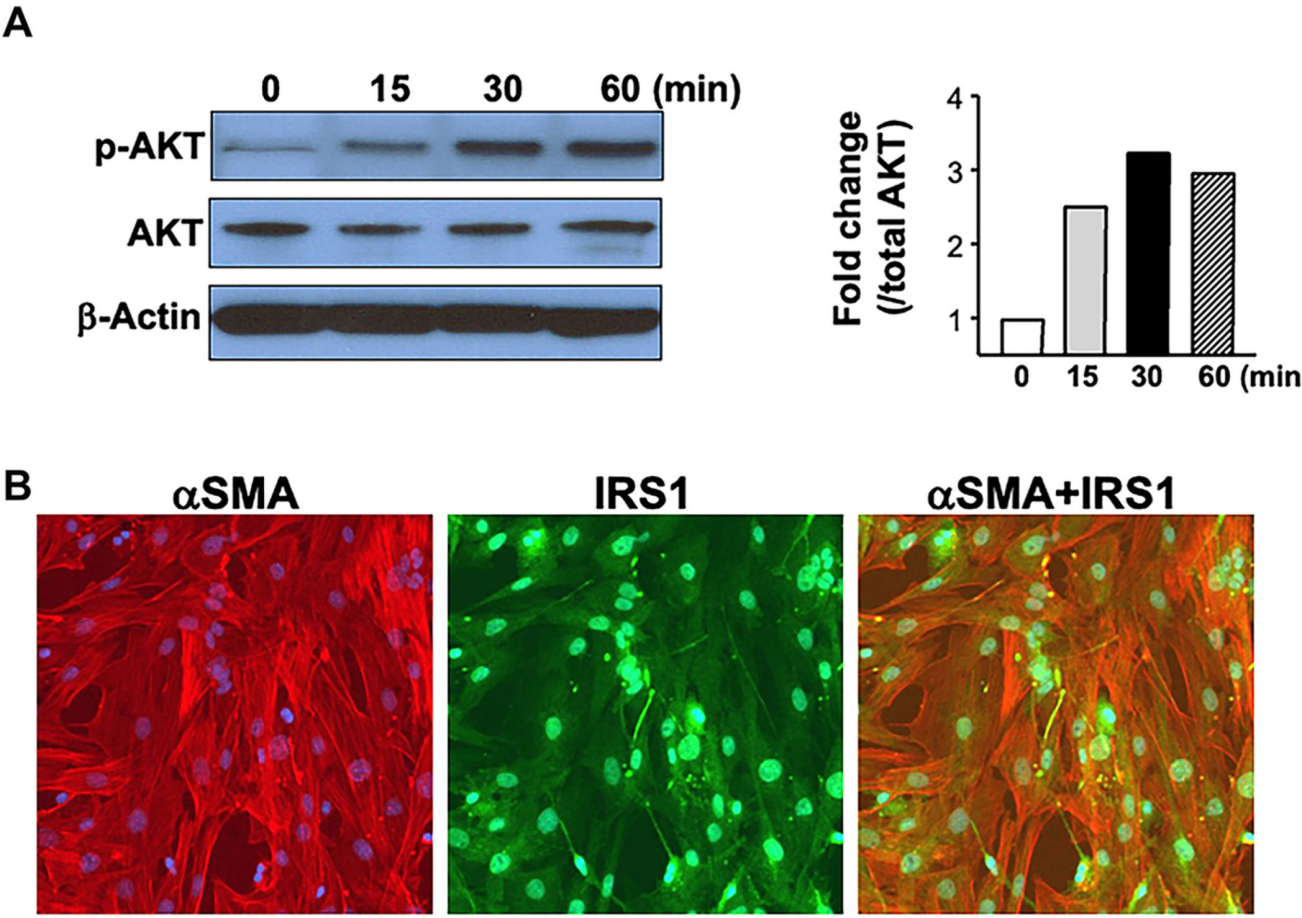
Insulin signaling in primary HSCs. (A) depicts AKT phosphorylation in response to insulin. Cells were treated with serum-free medium from days 5–7. On the 7th day, HSCs were treated with Ins (5 μmol) and the proteins were extracted at 15, 30, and 60 min of treatment. Western blot analysis of phosphorylated AKT (normalized to total AKT) showed induced AKT phosphorylation by Ins in HSCs. β-actin was used to demonstrate equal protein loading. Gels were scanned and the density of each band was divided by its corresponding loading Ctrl, and represented as fold change relative to time 0 in the accompanying graph; (B) demonstrates the presence of IRS1 in activated primary HSC cultures, detected by double immunofloresence. The last image shows the co-staining of IRS1 with α-SMA. HSCs: Hepatic stellate cells; AKT: protein kinase B; IRS1: insulin receptor substrate 1; α-SMA: alpha-smooth muscle actin; Ins: insulin; Ctrl: control.

Insulin signaling via the PI3 kinase pathway is active in HSCs, as evidenced by induced AKT phosphorylation by insulin treatment [Figure 4A]. Immunofluorescence analysis [Figure 4B] illustrates that α-SMA positive cells (activated HSCs) also express IRS1, one of the major substrates of the insulin signaling pathway.

**Figure 5.**
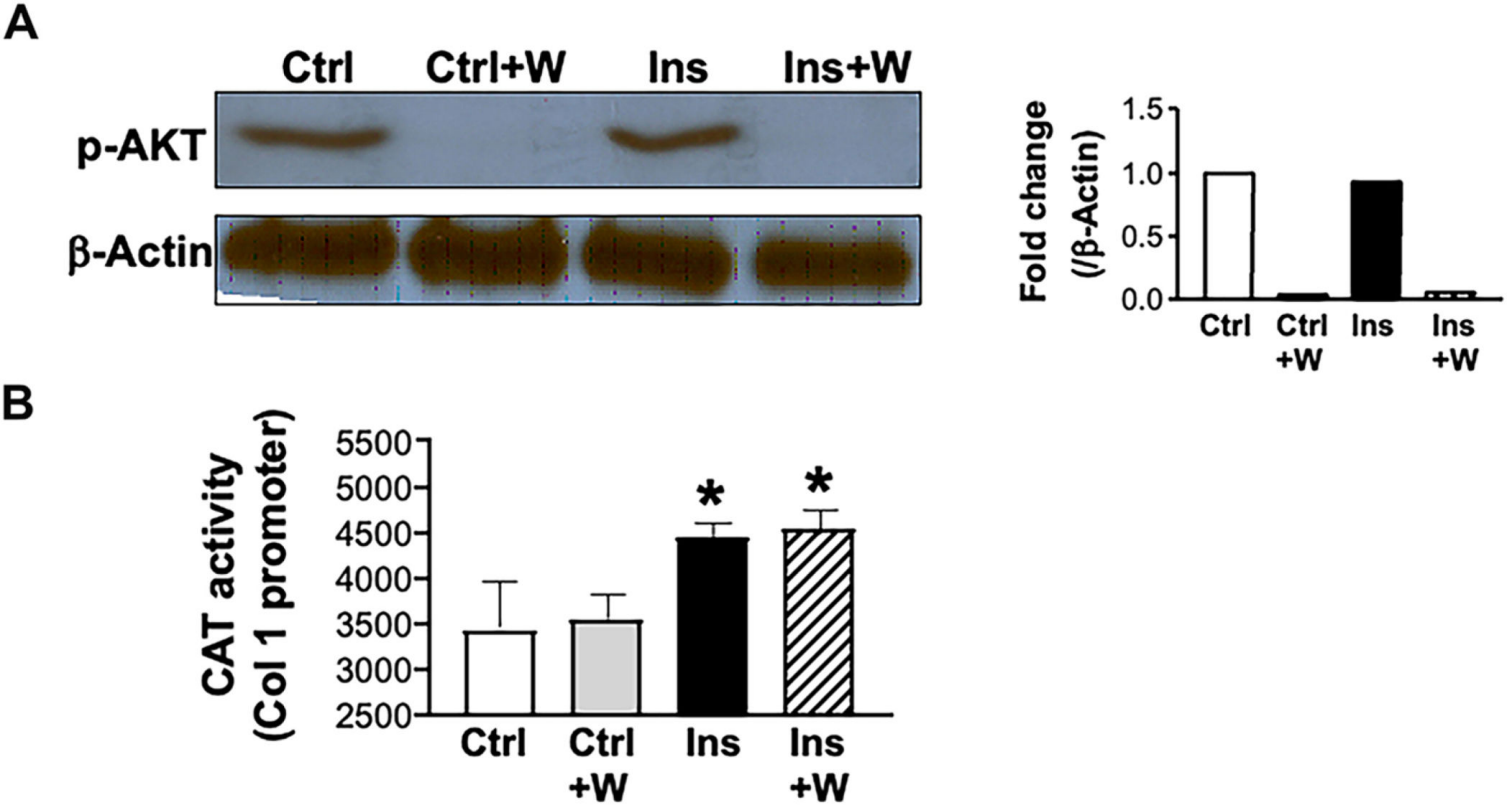
Insulin-induced collagen synthesis by HSCs is not mediated by the PI3 Kinase signaling pathway. HSCs were isolated from transgenic mice livers and plated as described previously. Cells were grown in high-glucose medium with or without Ins (5 μmol) and PI3 kinase inhibitor wortmannin (W) at a dose of 10 μmol/l. Cells were then harvested for protein extraction and CAT assay on day 7. (A) shows Western blot analysis of AKT phosphorylation by Ins in the absence but not in the presence of wortmannin. Gels were scanned and the density of the test band was divided by its corresponding loading Ctrl, represented as a fold change relative to Ctrl in the accompanying graph; (B) represents the CAT assay on day 7, showing no effect of wortmannin on Ins stimulation of COL1 promoter activity. HSCs: Hepatic stellate cells; CAT: chloramphenicol acetyltransferase; AKT: protein kinase B; Ctrl: control; Ins: insulin; COL1: type I collagen.

In order to determine whether the insulin effect on type I collagen production is mediated by PI3 kinase signaling, downstream of IRS1/AKT, HSCs were treated with wortmannin, a PI3 kinase inhibitor, in the absence or presence of insulin. Inhibition of PI3 kinase by wortmannin was demonstrated by abolished AKT phosphorylation in wortmannin-treated cultures [Figure 5A]. However, despite PI3 kinase inhibition, wortmannin treatment did not affect type I collagen promoter activity [Figure 5B], indicating that the insulin effect on HSCs is independent of PI3 kinase.

### Role of α5β1 integrin in insulin-induced type I collagen synthesis

**Figure 6.**
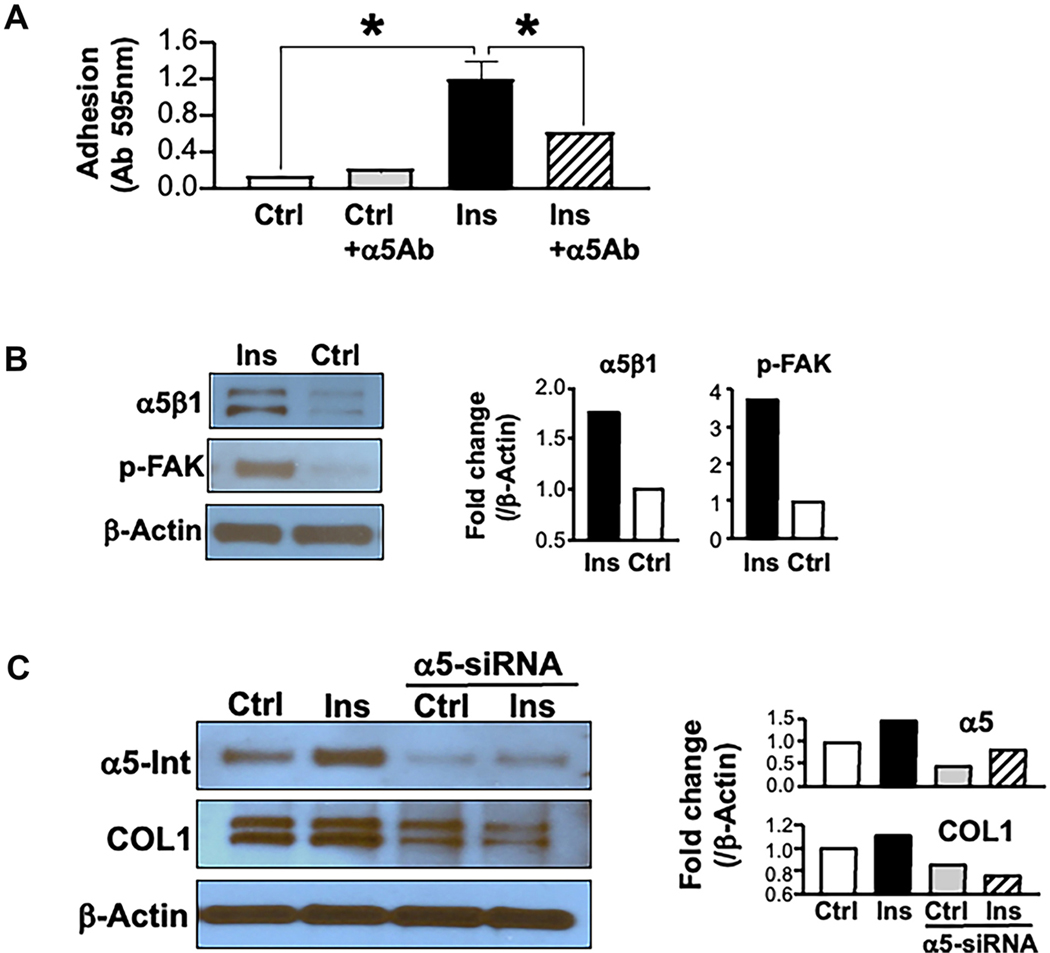
Insulin stimulates α5β1 integrin and its signaling pathway. (A) represents the adhesion assay in primary cultures of HSCs. Ins treatment (5 μmol) significantly increased HSCs adhesion to fibronectin and pretreating with α5Ab countered this effect, suggesting that increased adhesion is mediated by activation of α5β1 integrin; (B) shows Western blot analysis demonstrating increased levels of α5β1 integrin and p-FAK in Ins-treated HSCs; (C) shows that α5 siRNA inhibits the Ins effect on collagen synthesis in HSCs. Following siRNA-mediated downregulation of α5 integrin, cultures were grown in serum-free high glucose medium with Ins. Western blot analysis shows that Ins stimulation of both α5 integrin and COL1 synthesis was blocked in the absence of α5 integrin. HSCs: Hepatic stellate cells; Ins: insulin; α5Ab: α5β1 integrin blocking antibody; p-FAK: phosphorylated form of focal adhesion kinase; COL1: type I collagen.

As Figure 6A shows, insulin stimulated HSCs’ adhesion to fibronectin. Pretreatment with α5β1 integrin blocking antibody countered this effect, suggesting that increased adhesion is mediated through activation of α5β1 integrin. Western blot analysis showed increased levels of α5β1 integrin in parallel to increased FAK phosphorylation in insulin-treated HSCs [Figure 6B]. These findings suggest that insulin enhances the α5β1 integrin effect partly by increasing the number of available integrin molecules on HSC membranes, and partly by potentiating the α5β1 integrin signaling through FAK phosphorylation.

To further investigate if the insulin effect on type I collagen is α5β1 integrin-dependent, α5 integrin siRNA was used to lower α5β1 integrin in HSCs. As Figure 6C shows, knocking down α5 integrin in primary HSC cultures caused a decrease in type I collagen levels relative to controls (Ctrl). In addition, siRNA inhibited insulin-mediated increase in α5β1 integrin as well as type I collagen, suggesting that the profibrotic effect of insulin is, at least in part, mediated by an α5β1 integrin-dependent mechanism.

### Increased amount of ECM in livers and increased type I collagen synthesis by HSCs isolated from the hyperinsulinemic L-SACC1 transgenic mice

**Figure 7.**
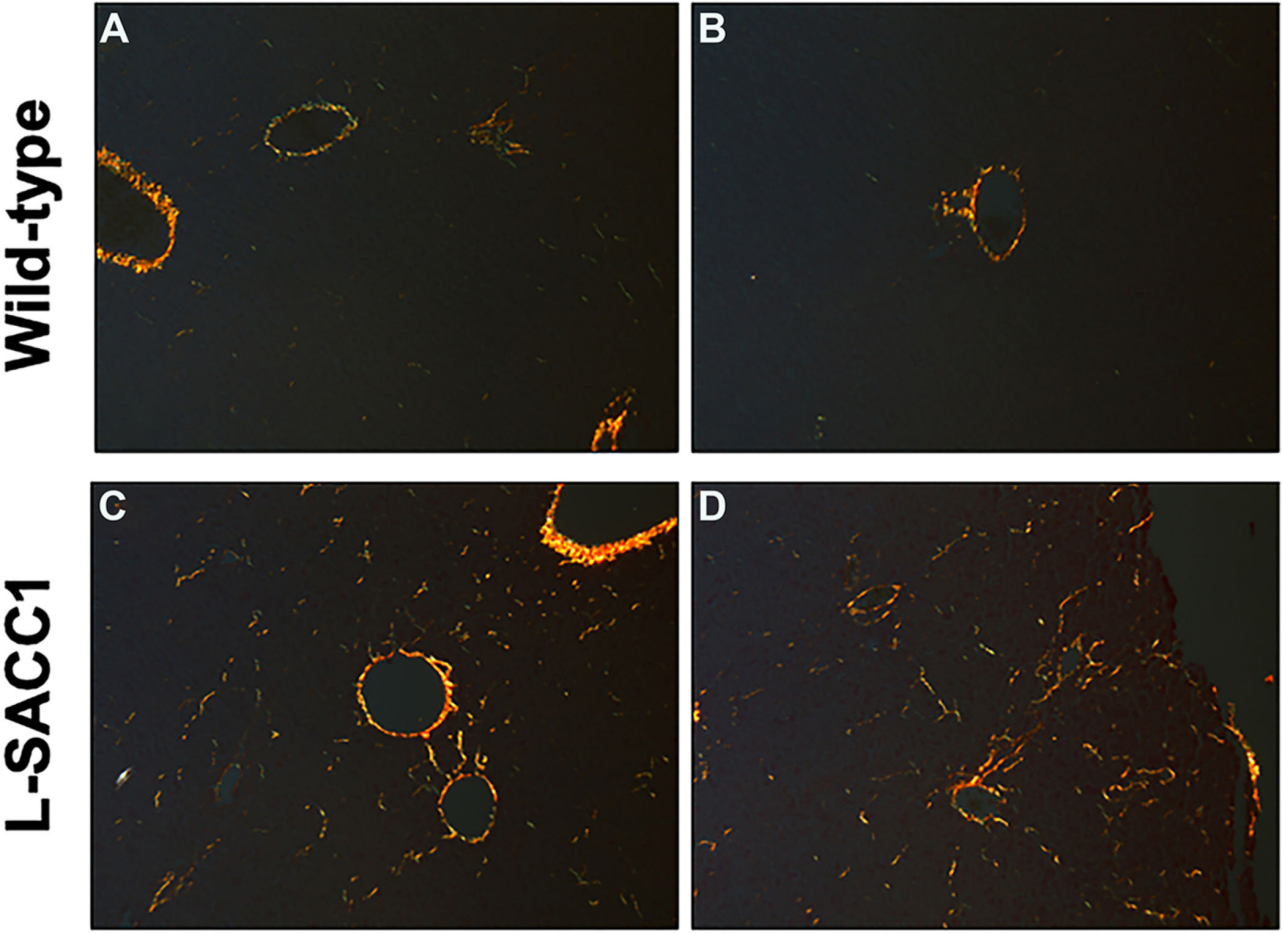
Type 1 collagen expression is increased in the livers of L-SACC1 transgenic mice. Sirius red staining (visualized under polarized light) of representative liver sections from wild-type (A and B) and L-SACC1 mice (C and D). L-SACC1 livers contain more stained birefringent COL1 fibers (seen as yellow on the black background). COL1: type I collagen.

Sirius red staining of random liver samples taken under baseline conditions showed increased deposition of ECM in the hyperinsulinemic L-SACC1 mice compared to wild-type. The increased amount of ECM is scattered throughout the acinus in a perisinusoidal fashion [Figure 7]. Image analysis on three randomly selected views of Sirius red-stained liver sections revealed that the total area of the slide stained for collagen was 0.27 ± 0.04% for wild type (*n* = 7) and 1.00 ± 0.15% for LSACC1 mice (*n* = 10, *P* = 0.000015).

**Figure 8.**
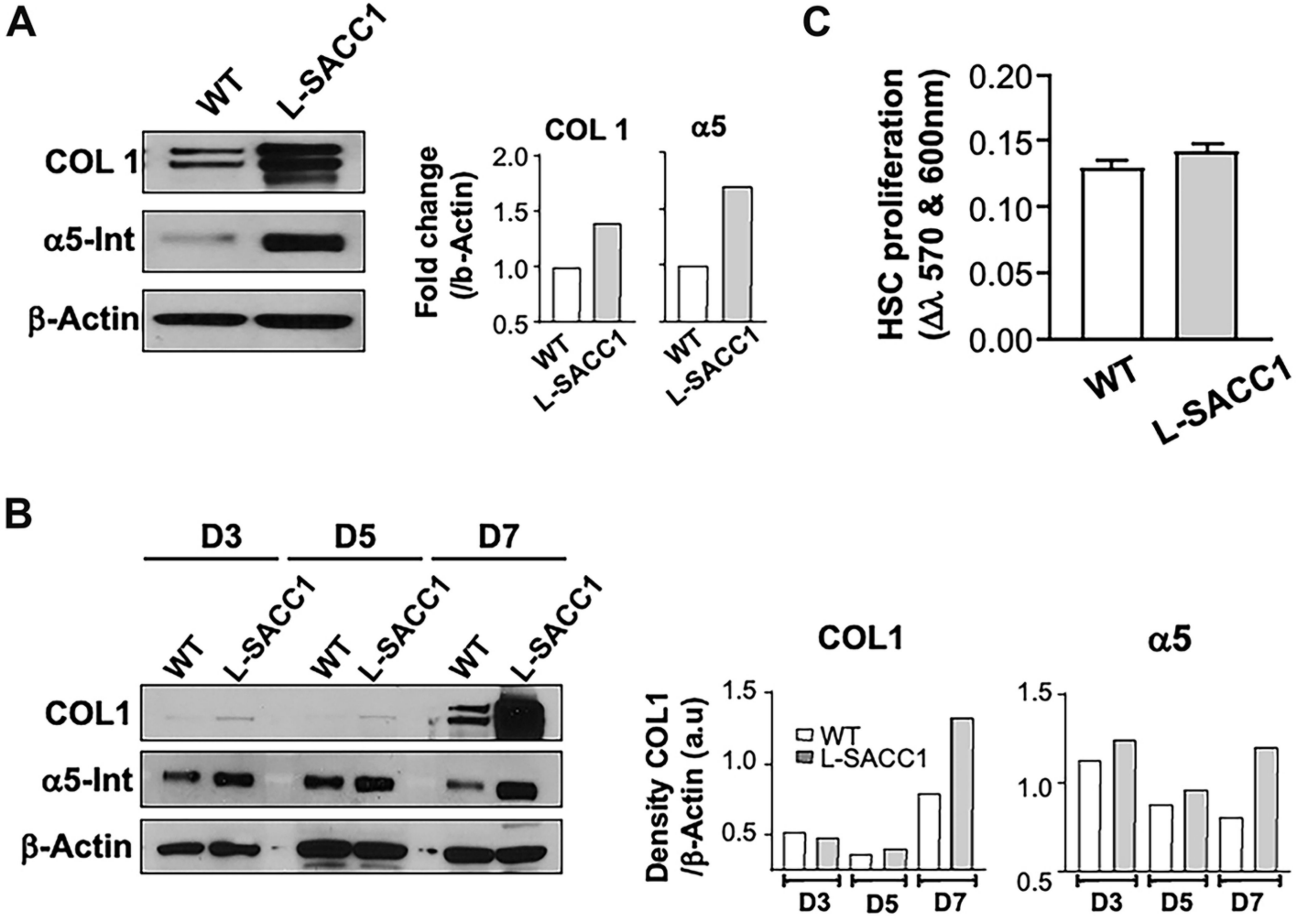
Elevated Type I collagen and α5β1 integrin levels in primary HSCs isolated from L-SACC1 mice. HSCs were isolated as described in Methods and cultured in DMEM with 450 mg/dL glucose, 10% FCS, and 10% HS. Cells were harvested at times indicated on individual panels for Western blot analysis and analyzed by Alamar blue assay for proliferation on day 6. (A) shows Western blot analysis demonstrating increased production of COL1 and α5 integrin by HSCs isolated from L-SACC1 mice compared to cells from wild-type animals; (B) represents a Western blot analysis on days 3, 5, and 7, showing significantly higher COL1 levels in L-SACC1 than in wild-type mice only on day 7. This suggests that HSCs from L-SACC1 mice do not undergo activation earlier in culture. In contrast, α5 integrin levels are increased even after 3 days in culture, with a more pronounced increase in HSCs from L-SACC1 compared to those from wild-type mice; (C) shows no difference in HSC proliferation in primary culture between L-SACC1 and wild-type cells. Cell proliferation was determined by a colorimetric change in the medium measured as a difference in culture media absorbance at 570 and 600 nm. HSCs: Hepatic stellate cells; DMEM: Dulbecco’s modified Eagle Medium; COL1: type I collagen.

HSCs isolated from L-SACC1 mice showed increased levels of type I collagen and α5 integrin on day 7 of culture [Figure 8A]. In addition, α5 integrin levels were higher in earlier cultures and showed no decrease on day 7 as seen in wild-type cells [Figure 8B]. Higher levels of type I collagen in HSCs isolated from insulin-resistant transgenic mice did not result from accelerated activation, based on no observed difference in type I collagen levels between insulin-resistant and wild-type cells in cultures earlier than Day 7 [Figure 8B]. Moreover, the proliferation of HSCs isolated from transgenic L-SACC1 mice was not significantly different from controls [Figure 8C], similar to what was observed in experiments with insulin treatment of primary HSC cultures.

## DISCUSSION

**Figure 9.**
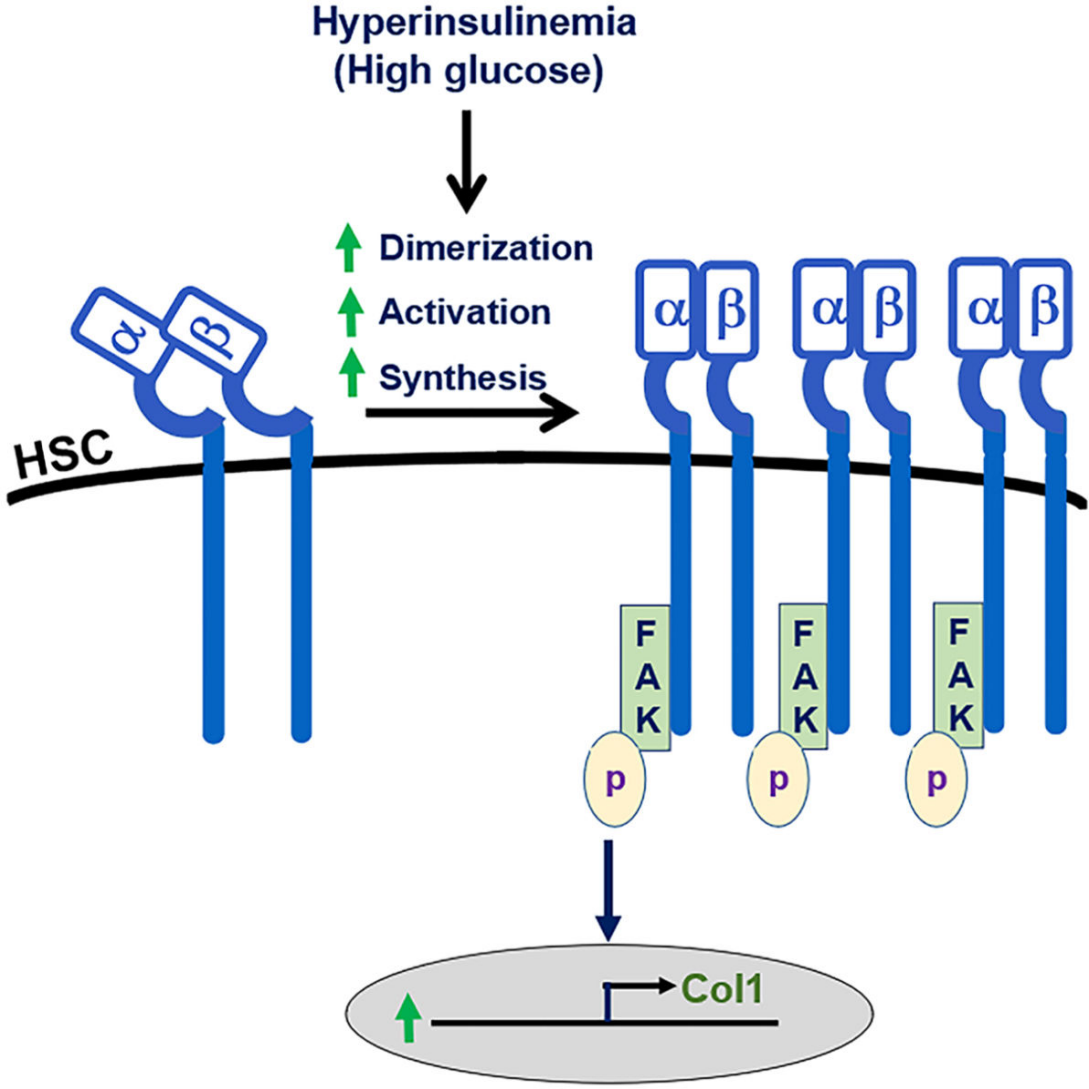
Proposed model of the effect of hyperinsulinemia and hyperglycemia on α5β1 integrin signaling and type I collagen synthesis. HSCs: Hepatic stellate cells; FAK: focal adhesion kinase.

Type 2 diabetes and insulin resistance are associated with advanced fibrosis and a worse prognosis, not only in MASLD but also in hepatitis C^[25]^. Direct hepatocyte injury by hyperinsulinemia and insulin resistance found in MASLD^[22]^ has been identified as important mediators of fibrosis progression in MASH^[12]^. Consistently, the current studies demonstrated that elevated insulin levels increase type I collagen production in HSCs, underscoring the direct profibrotic effect of hyperinsulinemia. This direct regulatory effect of insulin on type I collagen synthesis is independent of the PI3 kinase signaling pathway, but rather mediated by α5β1 integrin signaling. These observations are supported by previous reports on insulin potently activating α5β1 integrin signaling in CHO cells^[26]^, as well as the role of insulin interaction with α5β1 integrin in HSC activation^[24]^. Furthermore, blocking α5β1 integrin signaling by Arg-Gly-Asp peptides decreases liver fibrosis in both *in vivo* and *in vitro* experiments^[27,28]^. As depicted in the working model of Figure 9, the current studies show that hyperinsulinemia induces synthesis and dimerization (activation) of α5β1 integrin and subsequent propagation of its signaling via FAK phosphorylation, which in turn induces type I collagen synthesis.

Notably, the observed interaction with α5β1 integrin is not unique to insulin. Connective tissue growth factor (CTGF), basic fibroblast growth factor, insulin-like growth factor (IGF), and TGF-β are some of the other growth factors that have been reported to engage with α5β1 integrin in complex signaling^[29,30]^. Because IGF’s effects on HSCs are mainly mediated by the PI3 kinase pathway^[31]^, the data rule out a potential role for the IGF1 axis to mediate the stimulating effect of insulin on collagen synthesis and HSC activation. CTGF is a potent profibrotic factor involved in fibroblast proliferation, angiogenesis, and ECM synthesis^[32]^. Moreover, increased CTGF levels were reported in the livers of patients with MASH and animal models of type 2 diabetes, as well as in activated rat stellate cell cultures^[33]^. In the kidney, advanced glycation end products-induced CTGF expression, predominantly through a TGF-β1–independent pathway, plays a critical role in renal ECM accumulation^[34]^. However, we did not observe increased levels of CTGF in cultures treated with insulin (data not shown).

The mechanisms underlying the stimulatory effect of hyperinsulinemia on the activation of HSCs in insulin resistance and type 2 diabetes are not yet completely understood. Defects in the PI3 kinase signaling pathway have been implicated in the etiology of insulin resistance, ultimately leading to increased insulin levels in an attempt to overcome decreased intracellular signaling^[35,36]^. The resultant high insulin levels, however, can activate alternate signaling pathways, including α5β1 integrin signaling. The current data suggest that high insulin levels stimulate α5β1 integrin levels and phosphorylation of its downstream FAK signaling pathways. In *in vitro* systems, this mechanism is more pronounced in the presence of high glucose levels. The activation of stellate cells in the pancreas is augmented by both hyperinsulinemia and hyperglycemia^[37]^. Although other reports suggest that hyperglycemia alone can stimulate stellate cells^[38,39]^, incubating HSCs in media enriched with high glucose concentrations did not alter type I collagen promoter activity in the absence of insulin. The additive effect of high glucose levels on insulin may depend on the culture conditions. For instance, the other studies^[38,39]^ examined the effect of short exposure of stellate cells with a fully activated phenotype to high glucose. In contrast, we exposed primary cultures to high glucose throughout the entire culture period, starting in their quiescent phase and continuing through their activation process.

While the independent role of hyperglycemia in HSC activation awaits further analysis, it is worth mentioning that studies on the hyperinsulinemic normoglycemic transgenic L-SACC1 mice emphasize the dominant role of hyperinsulinemia in the activation of HSCs. CEACAM1 is a substrate of the insulin receptor, but not IGF-1R^[40]^ in the liver, and it upregulates receptor-mediated insulin endocytosis and degradation in a phosphorylation-dependent manner^[41]^. Consistently, L-SACC1 mice developed impaired insulin clearance and, consequently, hyperinsulinemia-driven insulin resistance without affecting pancreatic beta-cell function and insulin secretion^[15]^. Thus, L-SACC1 is a mouse model in which chronic hyperinsulinemia develops in the absence of fasting hyperglycemia and acts as a cause and not a consequence of insulin resistance^[15]^. Hyperinsulinemia in these mice develops in parallel to spontaneous hepatic fibrosis^[11]^. Consistently, the current studies demonstrate that their primary HSCs exhibit higher levels of type I collagen and α5β1 integrin than their wild-type counterparts, reinforcing the role of this integrin as a possible mediator of insulin-stimulated fibrogenesis. Interestingly, the dynamic of α5β1 integrin in L-SACC1 mice differs from that of their wild-type counterparts. As we have previously reported^[24]^, α5β1 integrin protein levels in HSCs isolated from wild-type mice rise at the earlier stages of activation and return to low levels once HSCs become fully activated in later cultures. In L-SACC1 HSCs, however, α5β1 integrin is higher early and throughout the time in cultures. Nonetheless, higher collagen synthesis by HSCs isolated from insulin-resistant mice cultured under the same conditions as wild-type cells suggests that the metabolic milieu, such as insulin resistance, may pre-program HSCs to respond by synthesizing more type I collagen once activation occurs due to liver injury. This would amplify the severity of fibrosis and its poor prognosis in conditions of chronic liver injury. In line with the concept of the “two-hit hypothesis” underlying the development of liver cirrhosis, hyperinsulinemia in insulin resistance may contribute to and facilitate the development of fibrosis after injury. This would, in a cumulative manner, increase scaring in the liver after every liver injury, and ultimately lead to clinically significant cirrhosis. Increased staining of the collagen matrix in L-SACC1 livers and in hepatocyte-specific CEACAM1 mutants^[12]^, as well as increased fibrosis in insulin-resistant animals subjected to fibrosis-inducing experimental conditions^[42]^, support this hypothesis.

### Strengths and weaknesses

The current studies provide an *in vivo* and *in vitro* demonstration that high insulin levels directly induce type I collagen synthesis and that this is mediated by α5β1 integrin/FAK signaling pathway. This novel finding is relevant in light of the mounting appreciation for the role of hyperinsulinemia in the pathogenesis of hepatic fibrosis in MASLD/MASH and T2DM^[22]^. Cell culture studies suggest that this effect of hyperinsulinemia is potentiated by the addition of high glucose concentrations. However, high glucose concentrations alone failed to induce the promoter activity of type I collagen. Together with the development of spontaneous hepatic fibrosis in these normoglycemic mice, this rules out a major role for hyperglycemia in the activation of HSCs. Nonetheless, further studies are needed to investigate the potentiating effect of hyperglycemia on HSC activation and progression of hepatic fibrosis, particularly in models of type 2 diabetes.

In summary, we postulate that high insulin levels directly induce collagen synthesis by HSCs via activating the α5β1 integrin/FAK signaling pathway. This suggests that in the hyperinsulinemic milieu of insulin resistance, HSC activation is augmented, leading to more robust fibrosis after injury in insulin-resistant patients with MASLD. Delineating the mechanisms that are responsible for the direct as well as the pre-programming effect of insulin on HSC activation may lead to new therapies that limit the rate of chronic liver disease and the need for liver transplantation in the ever-growing population of MASLD patients at risk.

## Data Availability

Original data for this study are available from the corresponding authors upon reasonable request.
